# Performance and profiling data of plane-wave calculations in quantum ESPRESSO simulation on three supercomputing centres

**DOI:** 10.1016/j.dib.2023.109614

**Published:** 2023-09-28

**Authors:** Worawan Marurngsith, Supakiet Waiphinit, Wiraporn Rosmode, Ruchipas Bavontaweepanya, Jiaxin Fan

**Affiliations:** a Department of Computer Science, Faculty of Science and Technology, Thammasat University (Lampang Campus) 248 M.2 Hang Chat, Lampang 52190 Thailand; b Thammasat University Research Unit in Quantum Technology, Thammasat University (Rangsit Campus) 99 Khlong Luang, Pathum Thani 12120 Thailand; c National Computational Infrastructure, Australia

**Keywords:** Performance evaluation, High performance computing, Quantum physics simulation, Profiling, Parallel and distributed computing, Catalytic reaction, Student competition, Message Passing Interface

## Abstract

This dataset reflects the parallel execution profiles of five Quantum ESPRESSO simulation (QE) versions in finding the total energy of the Cerium Oxide lattice using the self-consistent field (SCF) method. The data analysis used a strong scale setting to identify the optimal parameters and computing resources needed to complete a single SCF loop for one specific material efficiently. This analysis notably contributed to achieving the Best Performance Award at the 5th APAC HPC-AI Competition. The data comprises three sets. The first set features the parallel execution traces captured via the Extrae performance profiling tool, offering a broad view of the QE's model execution behaviour and how it used computational resources. The second set records how long QE's model ran on a single node at three HPC centres: ThaiSC TARA in Thailand, NSCC ASPIRE-1 in Singapore, and NCI Gadi in Australia. This set focuses on the impact of adjusting three parameters for K-point parallelisation. The final set presents benchmarking data generated by scaling out the QE's model across 32 nodes (1,536 CPU cores) on the NCI Gadi supercomputer. Despite its focus on a single material, the dataset serves as a roadmap for researchers to estimate required computational resources and understand scalability bottlenecks, offering general guidelines adaptable across different HPC systems.

Specifications Table

**Table:** 

Subject	Computer Science
Specific subject area	This paper concerns the execution performance analysis, profiling and benchmarking of scientific workload on high-performance computers.
Type of data	Tables, Paraver Trace Files, and CSV Files
How the data were acquired	The profiling and performance data were acquired by conducting performance evaluation experiments on three supercomputers all having CentOS Linux operating system. The experiments used the strong scaling approach to optimise the QE software's plane-wave function (pw.x) based on the same input lattice of Cerium Oxide (CeO_2_ having 20 k-points, N3=72 and NR3=96) under the same pseudo energy condition. All associate software was set up as following. • The pre-existing QE software package on various HPC systems was activated using the “module load” command. This included QE 6.5 on ThaiSC TARA, QE 6.6 and 6.7 on NSCC ASPIRE-1, and QE 7.0 on NCI Gadi. Additionally, a newer version, QE 7.1, was built into our project directory on the NCI Gadi system for explorative purposes regarding scalability. • Performance analysis tools pre-built from Barcelona Super Computing Centre, namely Paraver, Dimemas, Clustering, and Spectral, were installed in the project space on NCI Gadi. • The Extrae trace collection tool was configured and built from its source, linking with dependent libraries available in the NCI Gadi's environment. The installation was on our project directory. A dedicated folder was established to measure the performance data. The Ce.O_2_ input lattice was organised within the folder, accompanied by two pseudo-potential files. Each HPC centre's specific job scheduling system dictated the type of batch job scripts (*i.e.*, PBS for both Gadi and ASPIRE-1, and Slurm for TARA). These scripts informed the scheduler about job execution specifics. The job submission script detailed the following: • The request number of CPU cores, memory allocation, and estimated runtime. • Commands to configure the execution environment, establish parameter values (such as OMP_NUM_THREADS, NPOOL, NDIAG), load the QE software, and input the CeO_2_ data set with designated parameters. • The use of either “mpirun” or “srun” to initiate the parallel QE job on HPC compute nodes. These commands facilitate the Message Passing Interface (MPI) runtime, ensuring seamless communication across all CPU cores during the job. In the first part of the dataset, the execution trace collection was carried out on NCI Gadi to capture the comprehensive execution behaviour and pinpoint potential performance bottlenecks. The procedure for this collection is outlined below: • The LD_PRELOAD method was used to collect the trace files in this dataset. The method enabled Extrae to intercept MPI function calls, gathering execution data specifically for the pw.x function within QE. To facilitate this, the Extrae trace collection script, trace.sh, was inserted after the ‘mpirun’ command and before initiating the ‘pw.x’ command. • The information collected in trace files during the QE software's runtime adheres to a profiling specification written in the XML format (extrae.xml). • The traces from all CPU cores were saved temporarily in a directory named SET-0. Subsequently, these were merged into a raw trace file, TRACE.mpit. The raw trace file records time-stamped events generated by the QE execution.
	• Concluding the trace collection procedure, the mpi2prv command was invoked to transform the TRACE.mpit files, created by Extrae, into a .prv format. This format is compatible with and can be interpreted by the Paraver performance analysis tool. • Due to file size constraints, this dataset includes only the essential files required for visualisation using the Paraver performance analysis tool. These files encompass the .prv (Paraver trace file), a .row, and a .pcf (Paraver Configuration File) files. In the second and third parts of the dataset, the execution time of multiple QE runs was collected. Based on full-factorial experimental design, each run used a distinct parameter-level combination, leading to varying input lattice distribution patterns. Every execution was dispatched to the compute nodes using batch job submission systems. Upon completing each job, the execution profile was individually documented in separate Excel worksheets.
Data format	Raw, Analysed and Filtered
Description of data collection	Performance data was gathered to analyse and identify the optimal parameter settings in line with the 5th APAC HPC-AI Student Competition requirements, held online between June and October 2022 in the Asia Pacific region, including Australia. Each team received identical input files for the CeO_2_ lattice for the competition, accompanied by its pseudo-potential specifications. The central challenge was to conduct a strong scale analysis on the NCI Gadi supercomputer, aiming for the best execution time for the planewave function, which calculates the SCF loop for the provided input. Both the NCI Gadi and NSCC ASPIRE-1 supercomputers were made accessible to all competing teams. Each team was allocated the same amount of Service Units (SUs) and could utilise up to 32 48-core nodes in a single run. Though teams had the freedom to choose any QE version, version 7.0 was readily available on NCI Gadi. Teams could adjust three parameters: diagonalisation (NDIAG), number of CPU pools (NPOOL), and the number of OpenMP threads (OMP_NUM_THREADS). The use of a custom-built QE software stack was permissible. However, modifying any inputs or leveraging GPUs for acceleration was prohibited. The competition task required each team to conduct a single-node experiment to determine the optimal parameter settings before scaling out to multiple nodes. Given the permission to use a custom-build stack, we first gained insight into the QE execution dynamics before starting a single-node experiment. This was done by recording execution traces to visualise task distribution and pinpoint potential bottlenecks. Consequently, the first dataset includes these trace files, enabling a detailed execution trace visualisation through Paraver. Once these trace files are visualised within Paraver, one can thoroughly inspect the overall execution behaviour of QE. This provides a clear view of load imbalance issues arising from communication and computation, offering guidance on priority areas to enhance performance. A full-factorial experimental design was employed to determine the optimal parameter settings for executing the pw.x function on a single node. Since we also had access to the ThaiSC TARA supercomputer, having two QE versions, we conducted the single-node experiment across each QE version in three HPC centres. The second part of the dataset, “Screening performance study using a single computing node”, was gathered during this phase. Analysing this data revealed the dominant parameters consistently observed across all QE versions tested. Additionally, variation in execution times across diverse environments in different HPC centres was observed. From this dataset, the best parameter setting for optimal performance was identified.
	The best single-node parameter setting (OMP_NUM_THREADS=1, NPOOL=4 and NDIAG=9) was scaled out on the NCI Gadi multi-node environment. A critical new factor was finding the right number of nodes for k-point distribution and varying the “Process Per Resource” or PPR mapping available in the Open MPI implementation on the Gadi system. Again, a full-factorial experimental design was employed to observe each factor's impact systematically. The third part of this dataset, “Scalable performance data using multiple computing nodes” was collected during this process. The dataset includes the derived metrics against a single-core execution time as a baseline. The obtained speedup and Karp-flatt's metrics depicted the performance trend and underlined overhead.
Data source location	Institution: National Computational Infrastructure (NCI)’s Gadi System, hosted by the Australian National University City/Town/Region: Canberra, Australian Capital Territory (ACT) Country: Australia
	Institution: National Supercomputing Centre (NSCC)’s ASPIRE 1 System City/Town/Region: Connexis South Country: Singapore Institution: NSTDA Supercomputer Center (ThaiSC)’s TARA System City/Town/Region: Pathum Thani Country: Thailand
Data accessibility	Repository name: Mendeley Data Data identification number: 10.17632/6tp23c5dp7.2 Direct URL to data: https://data.mendeley.com/datasets/6tp23c5dp7/2
Related research article	-

## 1 Value of the Data

• Despite focusing on one material with a specific set of input parameters, the scaling analysis data depicts the general trends and identifies performance bottlenecks likely to be indicative of challenges faced in other computational setups. Thus, the data can assist material science researchers in estimating the required computational resources and the bottlenecks that impact their simulations.
• The execution data and profiling traces can complement larger datasets, contributing to developing models for HPC performance optimisation and anomaly detection.
• The dataset provides a template for conducting and presenting performance and scalability analysis. It offers general guidelines on how profiling data can be used to gain further insights into parallel execution and communication behaviour that could be adapted to different computational settings and systems.
• Experimental data and data collection methodology could be used for practical training on scalability analysis of parallel and distributed systems.
• The job submission scripts, input and output data, and build scripts could be used as a template for domain scientists to be adapted to different computational settings and systems.

## 2 Objective

**Fig. 1:**
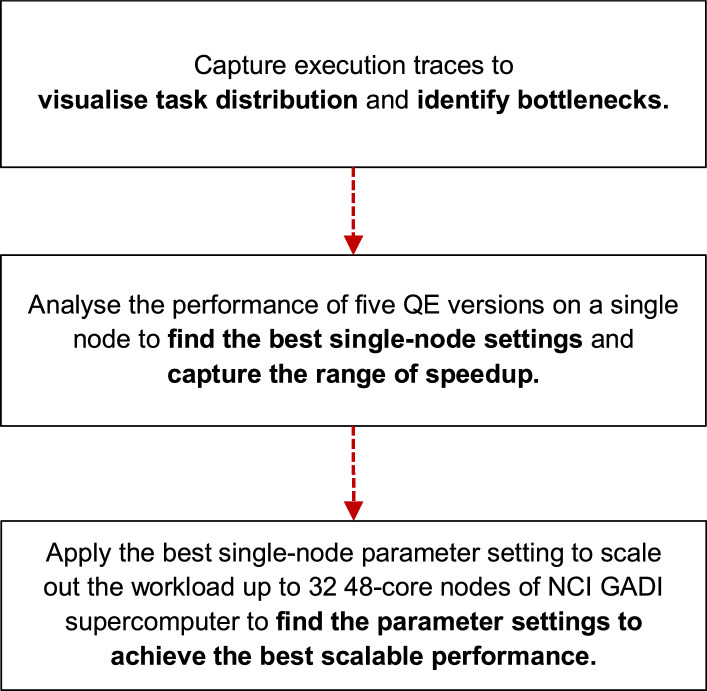
The systematic approach for dataset generation to meet the objective.

This dataset offers an exploration into the distributed processing behaviour of an SCF loop calculation in Quantum ESPRESSO software across various computational environments. The dataset was generated to respond to the 5th APAC HPC-AI competition aiming for optimal HPC performance. The K-point distribution was refined through detailed execution profile analysis; using a strong scaling setup, the goal was to identify the parameter setting towards the fastest execution time when expanding the workload with a consistent input set (CeO_2_ lattice) on the specific pw.x function. The dataset was realised through a three-step process shown in Fig. 1. Initially, the focus was visualising how the workload was distributed across multiple cores at run time to pinpoint inefficiencies. With that foundational knowledge, various versions of QE were tested on a single computational node on three HPC Centres: ThaiSC TARA Thailand, NSCC ASPIRE-1 Singapore, and NCI Gadi Australia, to identify optimal settings for maximal performance on that scale. Finally, having determined the best settings for a single node, those settings were then leveraged as a starting point for scaling the workload across a larger cluster of nodes. This scaling aimed to fine-tune parameters further to achieve the best performance when using the resource up to 32 48-core nodes’ environment of the NCI Gadi supercomputer. This data article provides the performance and profiling dataset used in the analysis, which other researchers can use to reproduce our results, perform case studies, or test different parameter tuning strategies.

## 3 Data Description

The dataset reflects the execution profiles and performance of calculating a plane-wave self-consistent field using the Quantum ESPRESSO simulation software [1,2] on HPC. Data was collected by launching batch jobs to the compute partition of the HPC centres using 1 to 1536 CPU cores; each job took the same input file and aimed to calculate the total energy of the Cerium Oxide lattice. The the exchange-correlation potential with the generalized gradient approximation (GGA) using the Perdew-Burke-Ernzerhof (PBE) exchange-correlation functional [9,10]. The interaction between electrons and ions was described by the Projector Augmented Wave pseudopotential. The valence electronic-structure wave function was expanded in the plane wave basis set. The kinetic energy cutoff for wavefunction was set to 50 Ry, and that for charge density was set to 400 Ry. The input lattice represents a finite number of points in the Brillouin zone called k-point mesh which can be manually partitioned for parallelly calculated by multiple CPU cores using the pw.x function. The k-point mesh was initially set to 20 in the calculation. The function distributes the partitioned mesh to CPU pools for iterative calculation using Message Passing Interface (MPI) and OpenMP-enabled libraries.

The dataset comprises three parts in the format of Paraver trace, and tabular form stored in spreadsheet files (.xlsx).

### 3.1 Detailed Execution Trace Visualised by Using Paraver

**Fig. 2:**
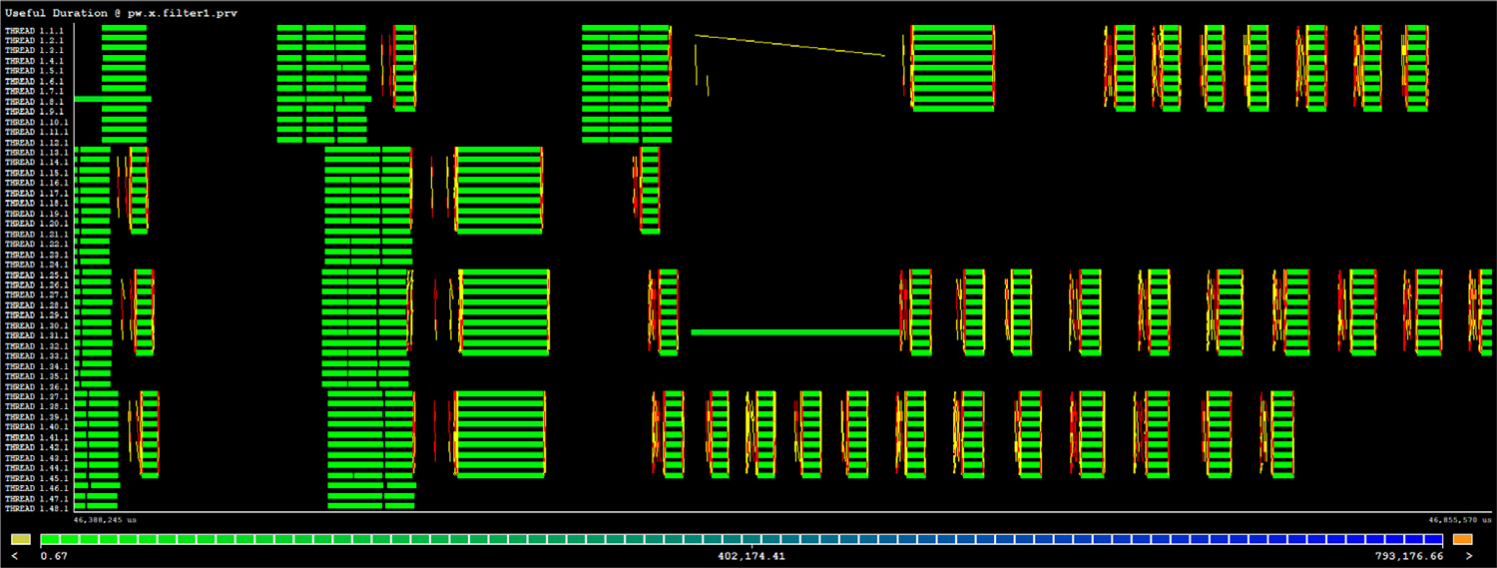
Visualization of MPI communication pattern amongst 48 MPI ranks showing heavy communication amongst diagonalisation threads (9 threads) in every thread pool (12 cores).

The first set is the single-node execution trace (.prv files) collected on a single node (48 CPU cores) of the NCIU Gadi supercomputer using the Extrae and Paraver tools [3]. These trace files must be opened by using the Paraver tool for interactively viewing different aspects of the QE's runtime activity. Table 1 and Fig. 2 below gives an example of performance information one could get from the trace files.

**Table 1:** Example of execution trace visualization and performance information generated by Paraver.

### 3.2 Screening Performance Study Using a Single Computing Node

**Fig. 3:**
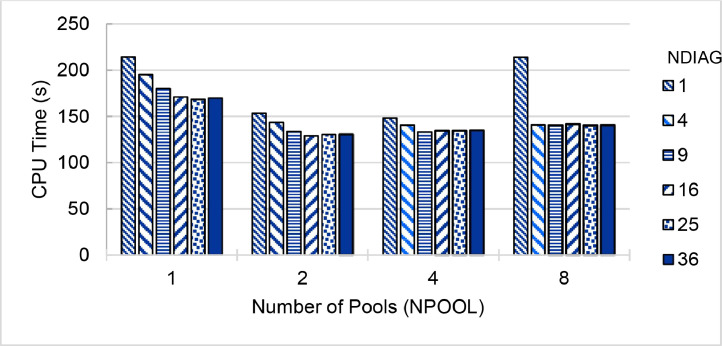
Run time of **QE 6.5 (pw.x)** on a single 40-core node of **ThaiSC TARA** Supercomputer, divided the CeO_2_ lattice k-points to 1 to 8 pools (NPOOL) and calculating diagonalization using 1–36 threads (NDIAG).

**Fig. 4:**
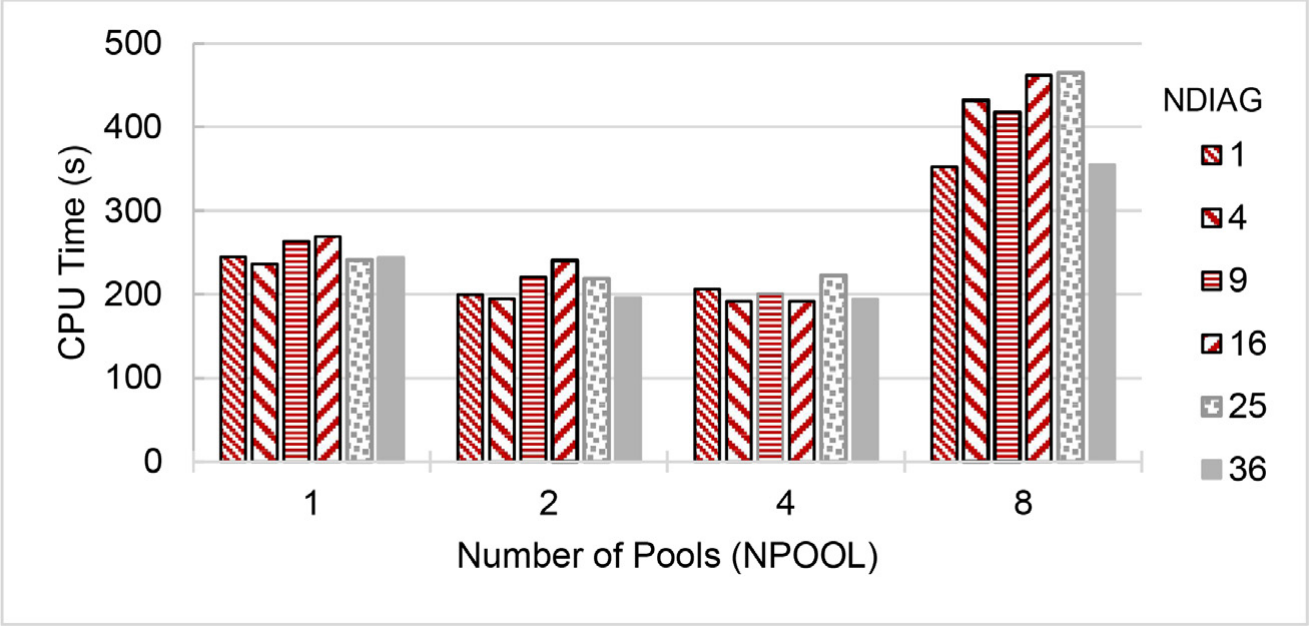
Run time of **QE 6.6 (pw.x)** on a single 24-core node of **NSCC ASPIRE-1** Supercomputer, divided the CeO_2_ lattice k-points to 1 to 8 pools (NPOOL) and calculating diagonalization using 1–36 threads (NDIAG). Greyed-out configurations over subscribe the available CPU cores.

**Fig. 5:**
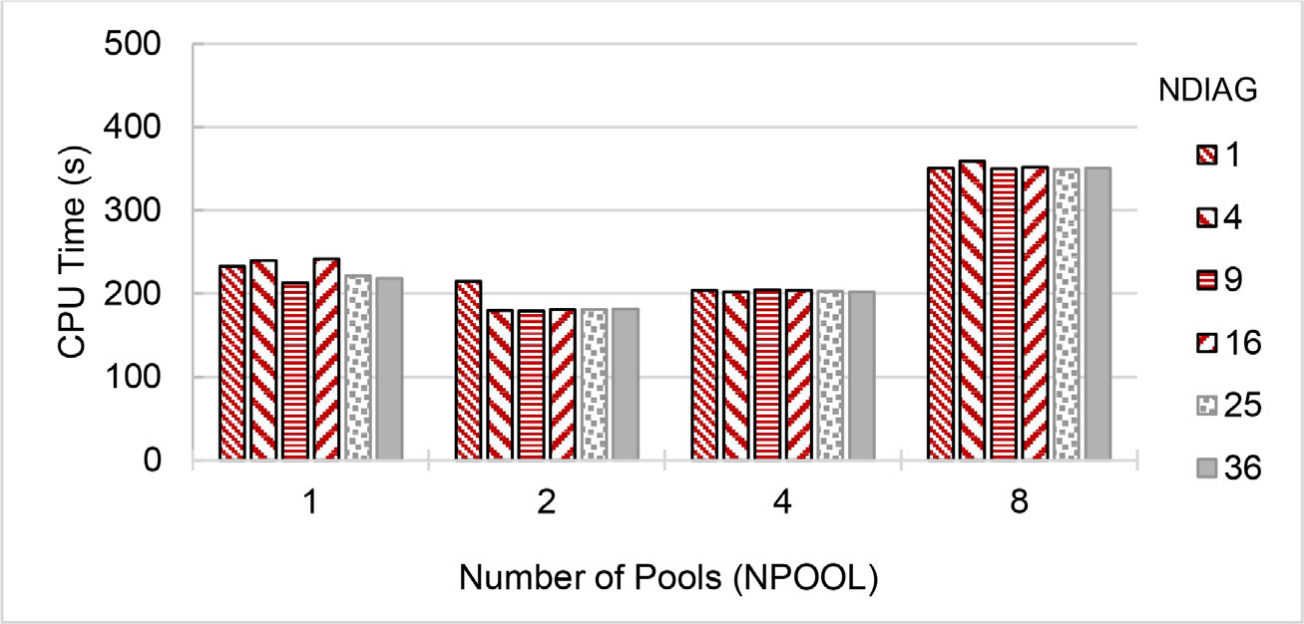
Run time of **QE 6.7 (pw.x)** on a single 24-core node of **NSCC ASPIRE-1** Supercomputer, divided the CeO_2_ lattice k-points to 1 to 8 pools (NPOOL) and calculating diagonalization using 1–36 threads (NDIAG). Greyed-out configurations over subscribe the available CPU cores.

**Fig. 6:**
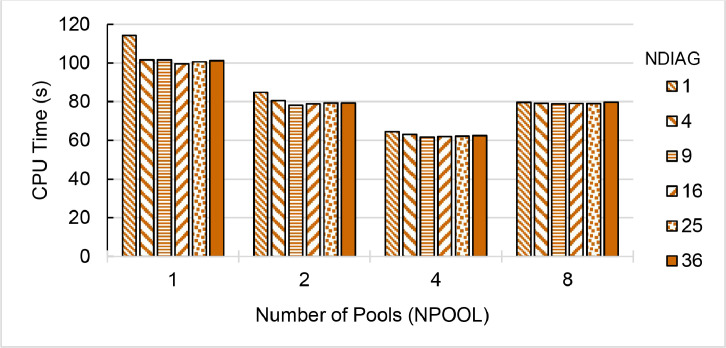
Run time of **QE 7.0-Native (pw.x)** on a single 48-core node of **NCI Gadi** Supercomputer, divided the CeO_2_ lattice k-points to 1 to 8 pools (NPOOL) and calculating diagonalization using 1–36 threads (NDIAG).

**Fig. 7:**
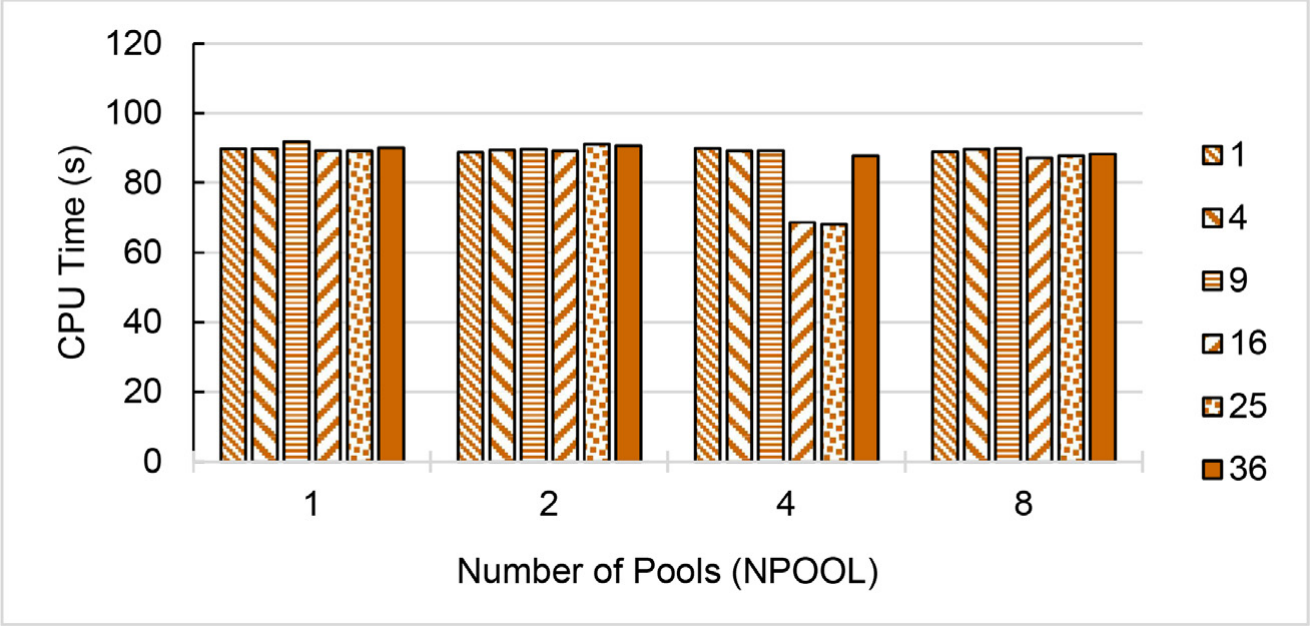
Run time of **QE 7.1 (pw.x)** on a single 48-core node of **NCI Gadi** Supercomputer, divided the CeO_2_ lattice k-points to 1 to 8 pools (NPOOL) and calculating diagonalization using 1–36 threads (NDIAG).

The second part of the dataset is the execution time and parallel speedup of a single node and 240 cores collected from ThaiSC TARA, NCI Gadi and NSCC ASPIRE1 supercomputers. The data presents in three spreadsheet files (.xlsx format) and could be used to create the following plots (shown in Fig. 3, Fig. 4, Fig. 5, Fig. 6, Fig. 7, Fig. 8) to analyses the single node performance. The broad range of execution time was observed to solve the same problem using the same number of CPU cores on different environments (shown in Fig. 9).

#### 3.2.1 Parallel execution time measured on a single node

#### 3.2.2 Parallel speedup

**Fig. 8:**
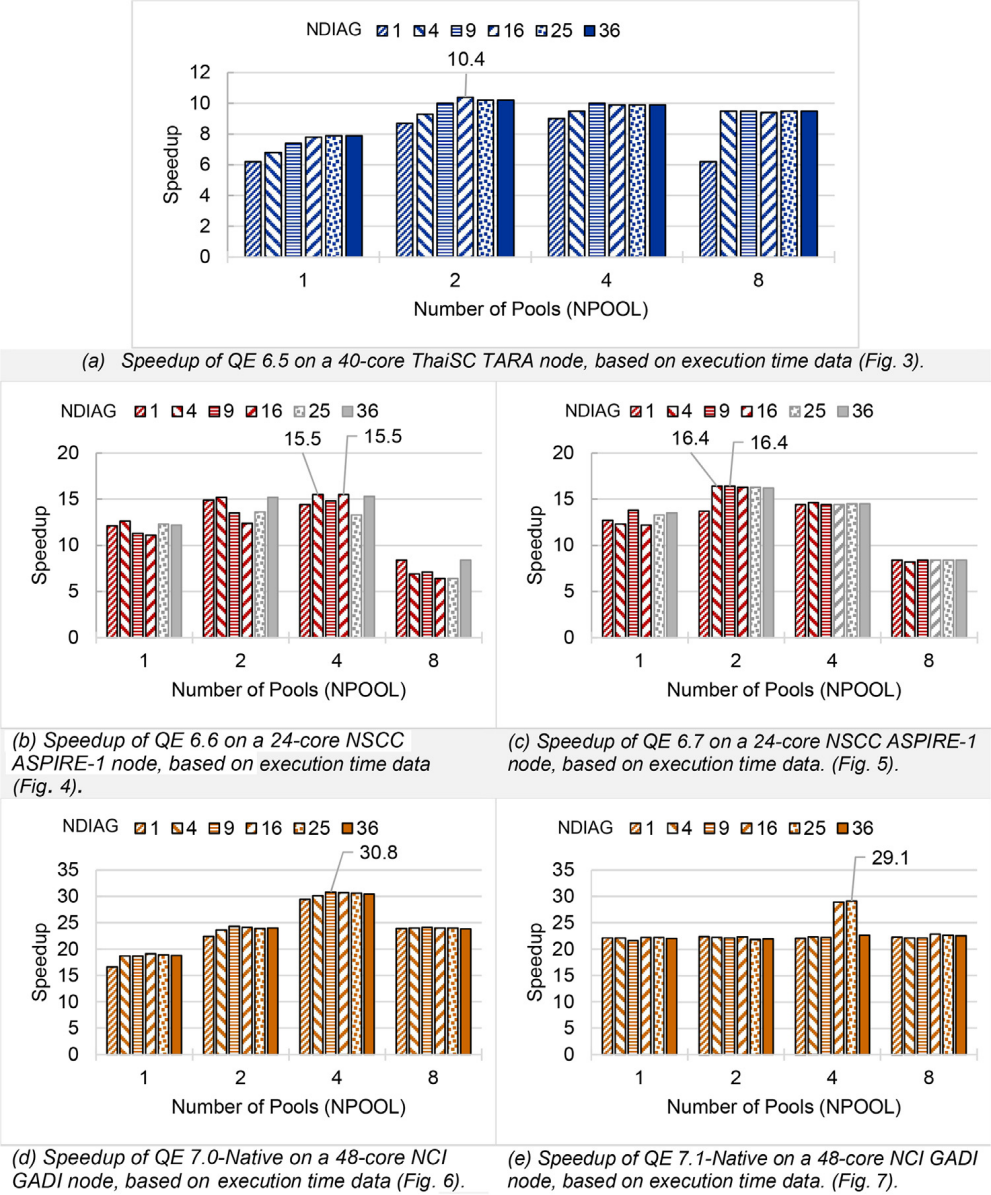
Single-node speedup of QE (pw.x) on (a) ThaiSC TARA (b-c) NSCC ASPIRE-1 (d-e) NCI Gadi divided the CeO_2_ lattice k-points to 1 to 8 pools (NPOOL) and calculating diagonalization using 1–36 threads (NDIAG). Performance is benchmarked against a single-core, single-pool, 1-thread diagonalization setup. Max speedup of each QE version is shown. Greyed-out configurations of the number of diagonalized threads oversubscribe available cores.

#### 3.2.3 Execution time of five QE versions running at 240 CPU cores on three HPCs

**Fig. 9:**
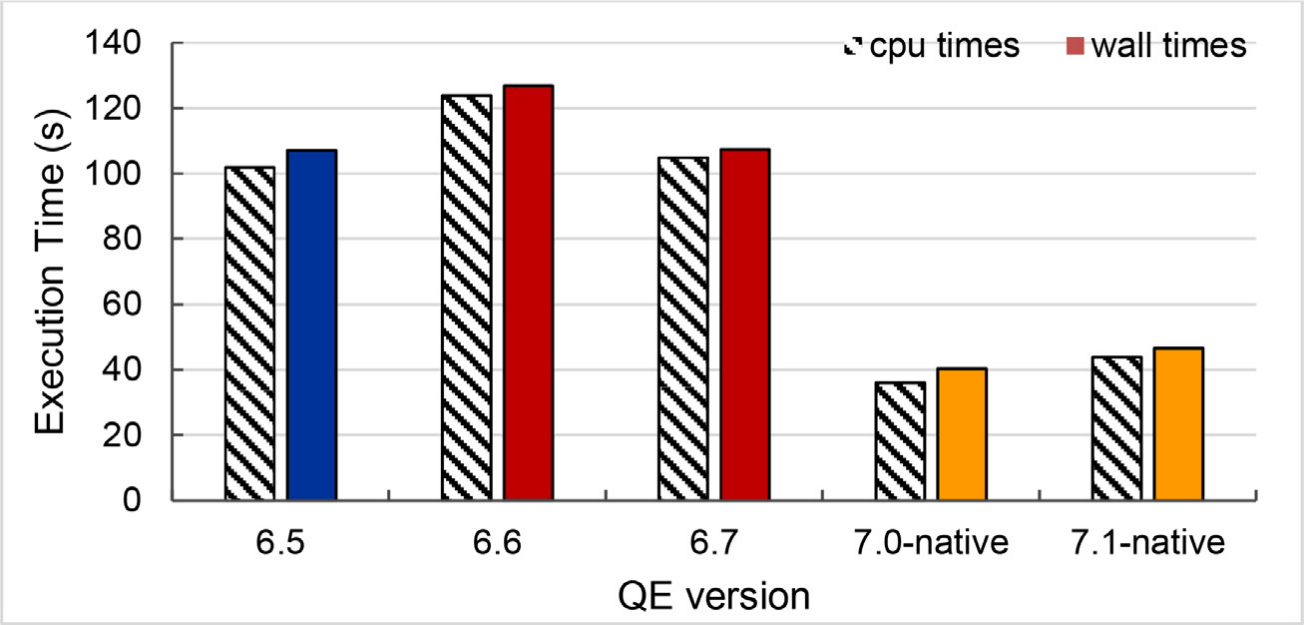
Execution time of calculating plane-wave on Ce.O_2_ lattice using 240 CPUs measured from QE 6.5 on ThaiSC TARA (Blue), QE 6.6 and 6.7 on NSCC ASPIRE-1 (Red), QE 7.0 and 7.1 on NCI Gadi (Orange).

### 3.3 Scalable Performance Data Using Multiple Computing Nodes

The third part of the dataset is the execution time and performance data collected when scaling the execution from 1 to 32 NCI Gadi supercomputer nodes, varying two parameters, i.e., NPOOL and NDIAG. The data presents in three spreadsheet files (.xlsx format) and could be used to create the following plots (shown in Fig. 10, Fig. 11, Fig. 12, Fig. 13) to analyses the scalability of the workload.

#### 3.3.1 Parallel execution time of QE 7.0 and 7.1 on NCI Gadi supercomputer

**Fig. 10:**
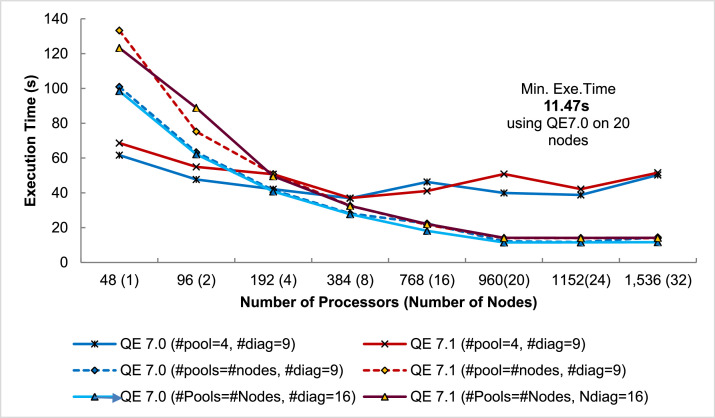
Execution Time of QE 7.0 vs QE 7.1 running PW.x (Ce.O2) on NCI Gadi Supercomputer measured on 1–32 48-core Gadi's nodes using three parameter settings. First, divided k-points into four pools (#pool = 4) and having 9 diagonal worker threads (#diag=9) was the best setting found in the single node experiment. The latter two settings was based on partitioning K-point to fit the number of nodes (#pool = #nodes). Two sizes of diagonal worker threads (#diag = 9 and 16) were explored. Best execution time observed shown in the plot (16-thread diagonalization).

#### 3.3.2 Parallel speedup of QE benchmarked against a single-core execution as baseline

**Fig. 11:**
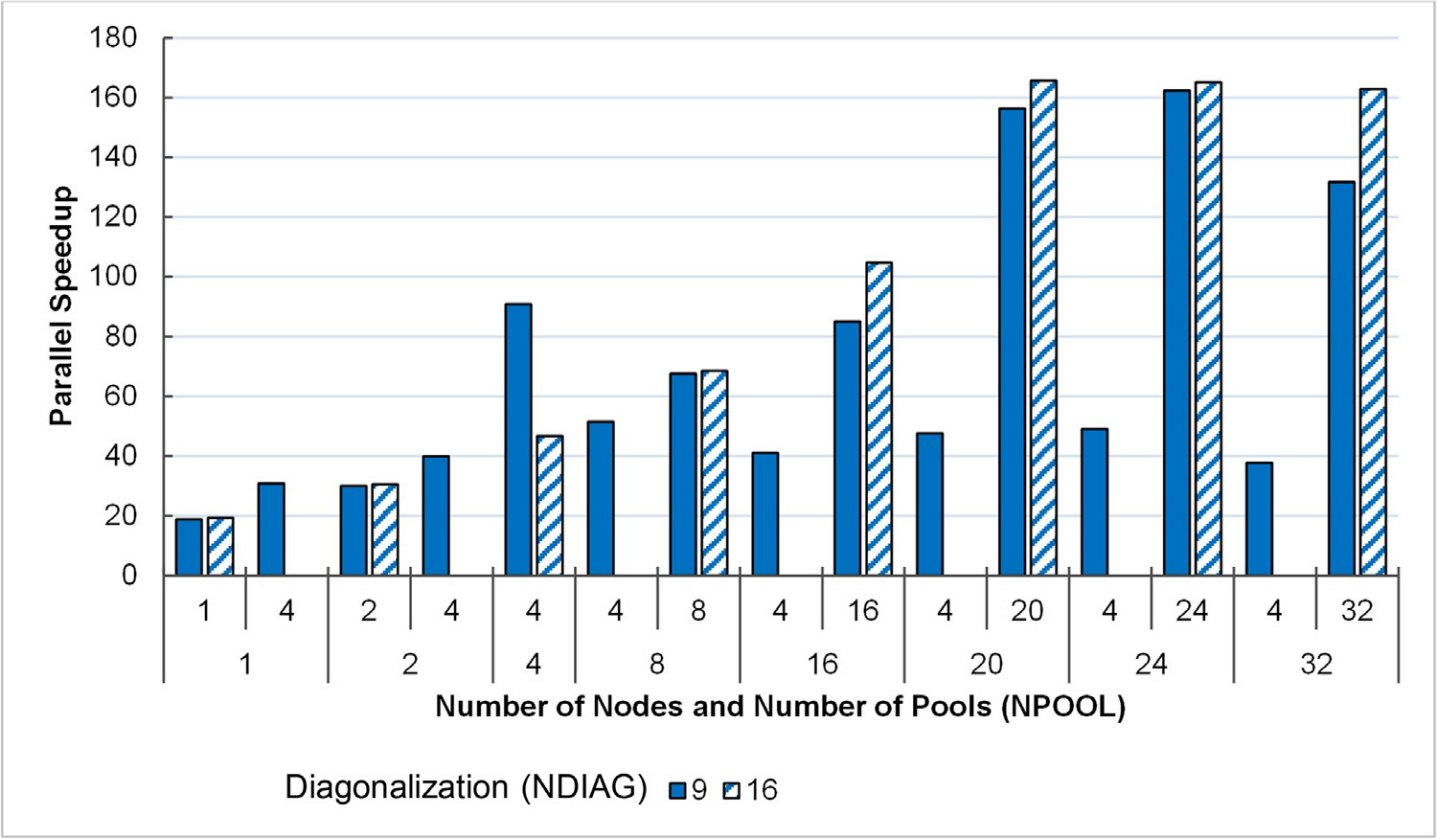
Parallel Speedup of QE version 7.0 measured on 1–32 48-core nodes of NCI Gadi supercomputer. Performance is benchmarked against a single-core, single-pool and 1-thread for diagonalization setup.

**Fig. 12:**
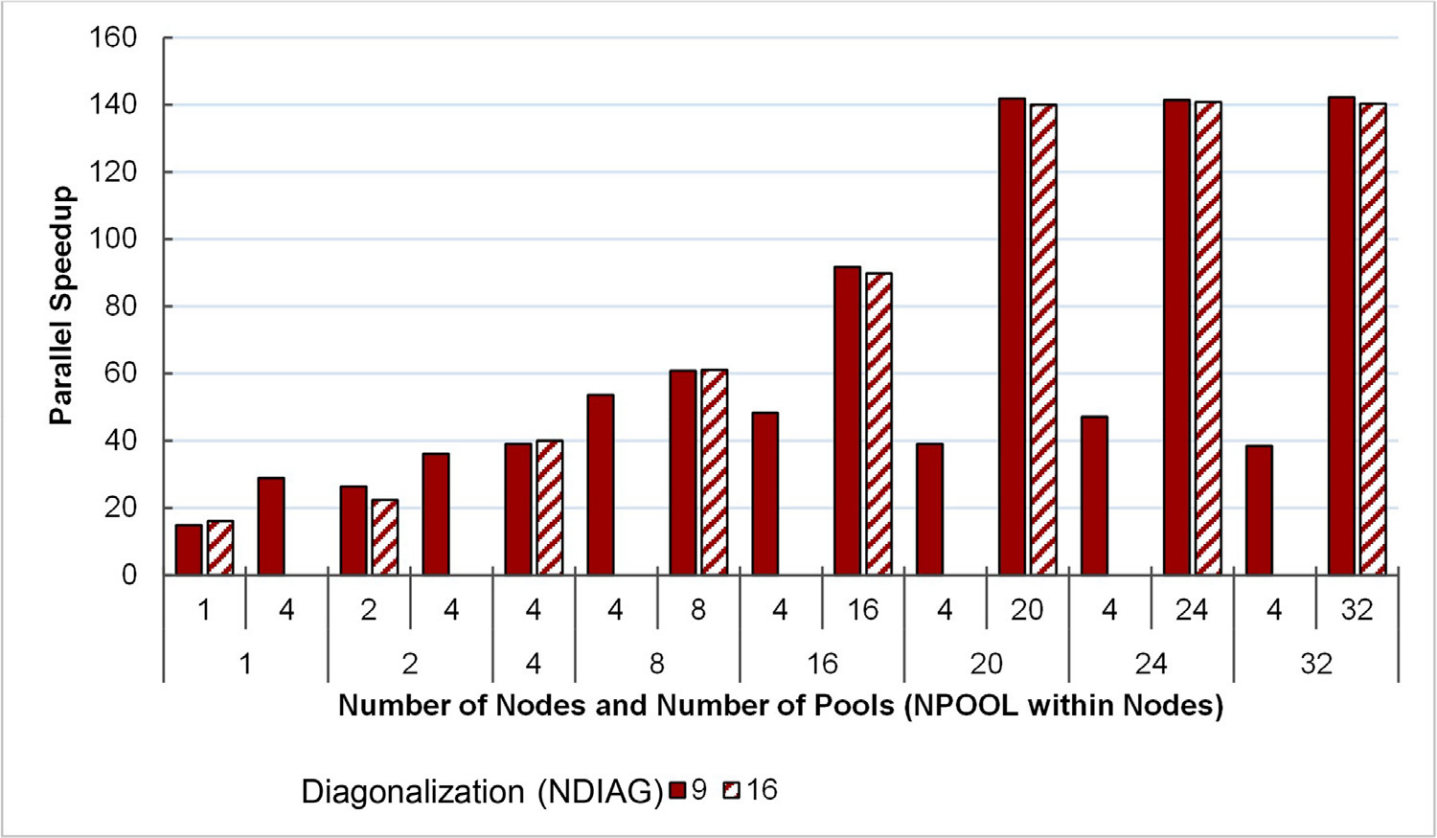
Parallel Speedup of QE version 7.1 measured on 1–32 48-core nodes of NCI Gadi supercomputer. Performance is benchmarked against a single-core, single-pool, and 1-thread for diagonalization setup.

#### 3.3.3 Scalability matric derived from speedup

**Fig. 13:**
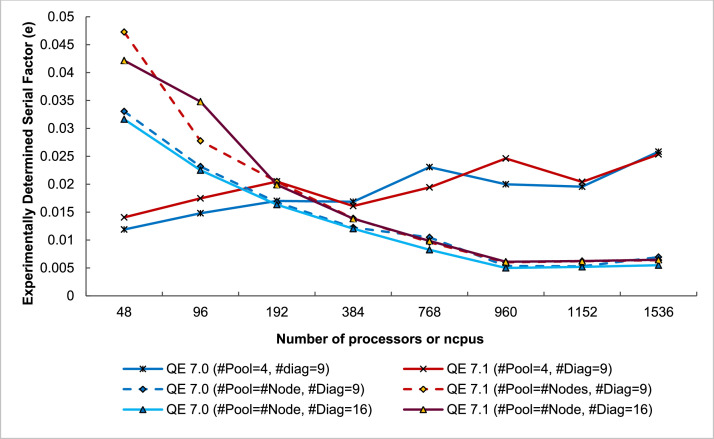
The Experimentally determined serial factor or Karp-flatt's Metric (e) depicted the overhead trend of QE 7.0 vs QE 7.1 running PW.x (CeO_2_) on 1 to 32 48-core nodes of NCI Gadi Supercomputer.

## 4 Experimental Design, Materials and Methods

### 4.1 Aims

This dataset collected specifically for the HPC benchmarking challenge at the 2022 APAC HPC-AI student competition [4]. The central goal of this challenge was to optimise the tuneable parameters to reach the optimal performance, *i.e.* the shortest CPU time for calculating the system energy on the NCI Gadi supercomputer [5]. Additionally, benchmarks were carried out on the NSCC ASPIRE-1 [6] and ThaiSC TARA [7] systems. This was to gain insight into the software structure and evaluate the performance impacts of tuneable parameters.

### 4.2 Materials

• **Access QE**. The QE software was access via loading module file on the HPC centres and via custom built the software from source code available on QE GitHub. This dataset has been collected using five versions of QE software (summarised in Table 3)
• **Job submission**
  ○ *input files*: CeO2.in – includes all the parameters to set up a SCF calculation using QE, Pseudopotentials – under the folder name /pseudo. The choice of pseudopotentials for Ce and atoms is based on the Standard solid-state pseudopotentials (SSSP).
  ○ *Submission script*: Jobs must be tested on a single node first then scale up to the maximum of 32 48-core nodes of the Gadi's normal queue. An example job scripts is as following.

**Table:** 

#!/bin/bash #PBS -q normal
#PBS -l walltime=00:30:00 #PBS -l ncpus=96 #PBS -l mem=100gb #PBS -l software=qe #PBS -P xx54 #PBS -o output-file.o #PBS -e error-file.e #PBS -l wd module purge module load qe/7.0 export OMP_NUM_THREADS=1 export nPool=4 export nDiag=9 echo "${PBS_NNODES}x${PBS_NCPUS}x${OMP_NUM_THREADS}-${nPool}-${nDiag}" > CeO2.out mpirun pw.x -npool ${nPool} -ndiag ${nDiag} -inp CeO2.in >> CeO2.out

• **Tunable and Non-Tunable Parameters**
  Parameter setup in the CeO_2_ energy calculation that were adjusted are number of pools (-npool), diagonalization (-ndiag) and number of OpenMP threads. Parameters that are directly associated with the model accuracy are NOT allowed to be modified, *i.e.,* The pseudopotentials and the input file (CeO2.in).
• **Platforms**
  The execution platforms and the computing resources provided by three HPC Centres (summarised in Table 2).

**Table 2:** Configuration of HPC Centres.

**Configuration**	**NCI Gadi**	**NSCC ASPIRE-1**	**ThaiSC TARA**
CPU	Inte Xeon Platinum 8268 CPU @ 2.90 GHz	Intel Xeon E5–2690 CPU @2.60 GHz	Intel Xeon Gold 6148 CPU @ 2.40GHz
Number of nodes	3074	1288	73
Number of cores per node	48	24	40
Number of nodes used in the experiments	1, 2, 4, 5, 8, 16, 20, 24, 32	1, 10	1, 6
Main Memory Configuration	192 GB RAM/node	128 GB DDR4 RAM/node	192 GB DDR4 RAM/Node
Interconnection	Infinitband HDR (200 Gbps) Dragonfly + topology	Infiniband EDR (100 Gbps) FAT Tree with full bisectional bandwidth within cluster	Infiniband EDR (100 Gbps) FAT Tree with full bisectional bandwidth within cluster
OS	Rocky Linux release 8.6 (Green Obsidian)	CentOS 6 Linux	CentOS 7 Linux
Job Submission System	PBS	PBS	Slurm
MPI used in the experiment	OpenMPI 4.1.4 built with Mofed 5.6 PBS 2021.1	Intel MPI 2019	Intel MPI 2018
• **Performance Analysis Software**
  The Extrae/Paraver performance profiling tool was installed on NCI Gadi and used for collecting execution trace. Fig. 13 shows the method to build the Extrae/Paraver tool and the steps to collect the single-node execution profiles of the QE workloads. The profiles were then visualised using Paraver to observe the change in runtime behaviour when setting different configurations.
• **Output**
  - Execution Traces
  - Some measured and derived performance metrics (summarised in Table 4) have been collected.

## 5 Experimental Design and Methods

**Fig. 14:**
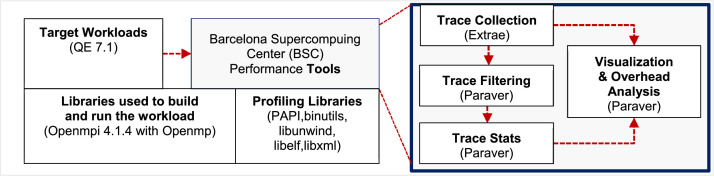
Method used to collect the execution trace (dataset 1).

The complete full-factorial experimental design has been used in studying single-node performance and multi-node scalability. Table 3, Table 4, Table 5 summarises the factor-level configuration, metrics and commands used for collecting execution time as reported in this data set. Five versions of QE software available and custom-built on three HPC centres (shown in Table 3) were run on every centre's compute node. The execution time of running the QE workload using a single-core, one pool and one diagonalization served as a baseline performance. Some derived performance metrics, i.e., speedup factor, efficiency and experimentally determined serial fraction (e) from Karp-flatt's metric [8] were calculated using the baseline execution time (Fig. 14).

**Table 3:** Experimental design for single node performance study.

Parameter No.	Tuneable Options	(1) QE 6.5 on TARA (40 CPUs)	(2) QE 6.6 on NSCC (24 CPUs)	(3) QE 6.7 on NSCC (24 CPUs)	(4) QE 7.0 on Gadi (48 CPUs)	(5) QE 7.1 +OpenMP on Gadi (48 CPUs)	(6) QE 7.1 no OpenMP on Gadi (48 CPUs)	(7) QE 7.1 Containerized on Gadi (48 CPUs)
1	OMP Threads	1, 2	1, 2	1	1	1	1	1
2	NPOOL	1, 2, 4, 8	1, 2, 4, 8	1, 2, 4, 8	1, 2, 4, 8	1, 2, 4, 8	1, 2, 4, 8	1, 2, 4, 8
3	NDIAG	1, 4, 9, 16, 25, 36	1, 4, 9, 16	1, 4, 9, 16	1, 4, 9, 16, 25, 36	1, 4, 9, 16, 25, 36	1, 4, 9, 16, 25, 36	1, 4, 9, 16, 25, 36
Total runs		96	64	64	96	96	24	24

Input: Ce.O2 Atom having 20 k-points, N3=72 and NR3=96.

Command: mpirun -np $PBS_NCPUS -x PATH=$PATH -x LD_LIBRARY_PATH=$LD_LIBRARY_PATH -display-map –mca btl ^openib /scratch/xx54/q-e-local/bin/pw.x -npool ${NPOOL} -ndiag ${NDIAG} -inp CeO2.in > CeO2.out.

**Table 4:** Derived and measured performance matrices used for comparing the results amongst HPC centres.

Baseline Performance Measurement	
Baseline execution time	ncpu=1, number of node=1, QE 7.0 and QE 7.1, NPOOL=1, NDIAG=1
Measured Sequential execution time (*T_s_*)	CPU Time (Second)
Metrics (Measured and derived)	Detail
Measured Parallel execution time on *p* CPUs (*T_p_*)	CPU Time (Second)
Parallel Speedup (ψ), derived	$\psi \;=\;\frac{T_{s}}{T_{p}}$

**Table 5:** Experimental design for multi-node performance study.

Experimental Factor	Levels
Number of Nodes (Number of CPUs)	1 (48), 2 (96), 4 (192), 8 (384), 16 (768), 20 (960), 24 (1152), 32 (1536)
QE Version	7.0 (Gadi's Module), 7.1 (Custom build with GNU Threads)
Model's Parameter	Setting
NPOOL	4, number of nodes
NDIAG	9, 16
Metrics (derived)	Detail
Karp-flatt's Metric (e) of *p* processors having ψ parallel speedup Experimentally determined serial fraction	$e\;=\;\frac{\frac{1}{\psi }-\frac{1}{p}}{1-\frac{1}{p}}$

## Ethics Statements

I declare that this submission follows the policies of Data In Brief as outlined in the Guide for Authors and in the Ethical Statement. This work does not involve studies with animals and humans.

## Declaration of generative AI and AI-Assisted Technologies in the Writing Process

During the preparation of this work the author(s) used GPT-4 in order to help improve the readability of the manuscript. After using this tool/service, the author(s) reviewed and edited the content as needed and take(s) full responsibility for the content of the publication.

## CRediT authorship contribution statement

**Worawan Marurngsith:** Conceptualization, Methodology, Writing – review & editing, Visualization, Supervision. **Supakiet Waiphinit:** Software, Investigation, Methodology. **Wiraporn Rosmode:** Software, Investigation, Data curation. **Ruchipas Bavontaweepanya:** Validation, Supervision, Writing – review & editing. **Jiaxin Fan:** Conceptualization, Resources.

## Declaration of Competing Interest

The authors declare that they have no known competing financial interests or personal relationships that could have appeared to influence the work reported in this paper.

## Data Availability

Performance and Profiling Data of Plane-wave Calculations in Quantum ESPRESSO Simulation on Three Supercomputing Centres (Original data) (Mendeley Data).
